# Comparative Analysis of the Characteristics of Triterpenoid Transcriptome from Different Strains of *Wolfiporia cocos*

**DOI:** 10.3390/ijms20153703

**Published:** 2019-07-29

**Authors:** Guiping Zeng, Zhong Li, Zhi Zhao

**Affiliations:** Guizhou Key Laboratory of Propagation and Cultivation on Medicinal Plants, Guizhou University, Guiyang 550025, China

**Keywords:** *Wolfiporia cocos*, triterpenoid, transcriptome, differentially expressed genes

## Abstract

The dried sclerotia of *Wolfiporia cocos* (Schwein.) Ryvarden & Gilb., a traditional Chinese medicine, has triterpenoid as its main active component. Breeding high-yield triterpenoid in *W. cocos* is an important research topic at present. We screened out two monosporal strains from the same *W. cocos* 5.78, high-yielding DZAC-Wp-H-29 (H) and low-yielding DZAC-Wp-L-123 (L), and cultured mycelia for 17 days, 34 days, and 51 days, respectively. Transcriptome analysis results showed that triterpenoid synthesis is closely related to gene expression in triterpenoid synthesis pathways (hydroxymethyl glutaryl-CoA reductase (HMGCR), farnesyl diphosphate synthase (FDPS), 4-hydroxybenzoate polyprenyltransferase (COQ2), C-8 sterol isomerase (ERG2), sterol *O*-acyltransferase (ACAT), tyrosine aminotransferase (TAT), torulene dioxygenase (CAO2), and sterol-4alpha-carboxylate 3-dehydrogenase (erg26)), and is limited by the expression of enzyme M20 combined with domain protein peptide (Pm20d2), aryl-alcohol dehydrogenase (norA), ISWI chromatin-remodeling complex ATPase ISW2, GroES-like protein (adh), cytochrome P450 (ftmP450-1), and unknown proteins unigene0001029 and unigene0011374. In addition, maintaining high triterpenoid accumulation in *W. cocos* may require a stable membrane structure, so the accumulation ability may be related to the high synthesis ability of sterols. The low accumulation of triterpenoid in *W. cocos* may be due to the products of key enzymes increasing flow to other pathways.

## 1. Introduction

*Wolfiporia cocos* (Schwein.) Ryvarden & Gilb, belongs to the genus *Wolfiporia* of the family Polyporaceae (Basidiomycota: Agaricomycetes: Polyporales) [1]. Wild *W. cocos* are distributed in East Asia, Australia, the United States, and other regions [2]. Most of the plants are parasitic in the roots of Masson pine or Pinus densiflora. In traditional Chinese medicine, the dry sclerotium of *W. cocos* (Hoelen in Japan) has a history go back more than 2000 years. It has the effects of diuresis, invigorating the spleen, and tonifying, tranquilizing the heart, and soothing the spirit. It is used for urinary problems, phlegm, dizziness and palpitations, spleen deficiency, loose stools, restlessness, and insomnia [3]. *W. cocos*, an important raw material for nearly 300 kinds of Chinese patent medicines, was called “four-time divine medicine” by the ancients [4]. It can also be made into a variety of health foods, cosmetics, etc. [5,6,7]. Modern studies have shown that triterpenoids in *W. cocos* have diverse pharmacological effects, such as anti-epilepsy, anti-apoptosis, diuresis, cytotoxicity, liver protection, anti-leukemia, anti-inflammatory, and so on [8,9,10,11,12,13,14,15,16]. Therefore, *W. cocos* has a wide and important role in future medical application.

The main active components of *W. cocos* are polysaccharides and triterpenoids; it also contains ergosterol, gum, chitin, protein, fat, sterol, lecithin, dextrose, etc. [17]. Triterpenoids are natural compounds with complex and diverse structures that are derived from the polymerization of isoprene. In the past two decades, Japanese and Chinese researchers have basically isolated almost all the triterpenoids from *W. cocos*, which are derivatives of lanosterol or secondary lanosterol [17].

Most of the research on *W. cocos* has focused on compositional analysis and its pharmacological action. There are few studies on its molecular biology, especially molecular genetics. Zhang et al. [18] sequenced the transcripts of the mycelium and early sclerotia of *W. cocos*, and found that in the early stage of sclerotial growth, carbohydrates activate enzymes including glycosyl hydrolases (GHs), carbohydrate esterases (CEs), auxiliary activities (AAs), carbohydrate-binding modules (CBMs), glycosyltransferases (GTs), and polysaccharide lyases (PLs) involved in carbohydrate metabolism. Part of the upregulated enzyme genes in the early stage of sclerotial growth may be involved in the formation of sclerotia in *W. cocos* parasitizing pine. He et al. [19] analyzed simple sequence repetition (SSR) in the transcriptome of *W.cocos* and the molecular function of these SSR-containing genes. It was found that the types of SSR in *W. cocos* were rich and had high polymorphism potential. Wu et al. [20] sequenced the transcriptome of early, middle, and mature stages of *W. cocos* sclerotia and found that gene expression patterns in the mature stage were significantly different from those in the other two stages. Shu et al. [21] sequenced the transcriptome on mycelia and sclerotia of *W. cocos* and revealed that triterpenoids were only derived from the mevalonate pathway (MVA), and found that diphosphomevalonate decarboxylase (MVD), farnesyl diphosphate synthase (FDPS), hydroxymethyl glutaryl coenzyme A reductase (HMGCR), and lanosterol synthase (ERG7) gene expression was upregulated in the hyphal stage, and it was speculated that these genes were involved in triterpenoid biosynthesis in the hyphal stage.

These results provide a good foundation for mining and researching functional genes of *W. cocos*, but the biological gene expression and the synthesis of secondary metabolites follow the order of time and space, they do not analyze gene expression over a time. The difference between mycelium and sclerotia in Shu et al. [21] only shows the difference of gene expression in different stages of development of *W. cocos*, but the difference in expression and regulation mode of genes related to triterpenoid synthesis in mycelium or sclerotia are still unclear.

The purpose of this study is to reveal the key genes related to triterpenoid biosynthesis and its regulation factor in mycelia of *W. cocos*. We screened out two monospore strains as the experimental material, which were significantly different in accumulation of total triterpenoid from the sexually reproduced progeny of *W. cocos* 5.78 (purchased from the Institute of Microbiology, Chinese Academy of Sciences, Beijing, China). Three culture times were chosen for mycelium transcriptome analysis, which had significant differences in the period accumulation of total triterpenoid. Our aims are to identify key genes and regulatory factors related to triterpenoid biosynthesis, and lay a theoretical foundation for molecular-assisted screening and breeding of *W. cocos*.

## 2. Results

### 2.1. Differences in Total Triterpenoid Content between H and L

**Figure 1:**
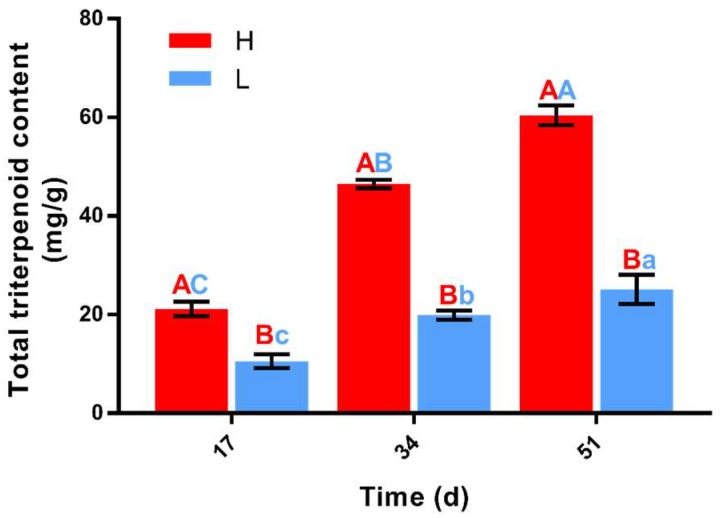
Total triterpenoid content at different culture times in high-yielding (H) and low-yielding (L) strains. Red letters indicate significant differences in least-significant difference (LSD) detection at the same time point between the two strains. Blue letters indicate significant differences between time points for the same strain. Capital letters indicate highly significant differences, and lowercase letters indicate significant differences.

The content of total triterpenoid in *W. cocos* was measured by the colorimetric method [22]. After the two strains were cultured for 17 days, the average total triterpenoid contents was 21.18 mg/g and 10.58 mg/g in high-yielding (H) and low-yielding (L) strains, respectively; at 34 days, the content was 46.51 mg/g and 19.91 mg/g, and at 51 days it was 60.41 mg/g and 25.16 mg/g in H and L, respectively. The results show that the accumulation of total triterpenoid was significantly different among different strains (Figure 1). The total triterpenoid content in H was highly significantly different at the three culture times. The total triterpenoid content in L was highly significantly different between 17 and 34 days, and between 34 and 51 days. The ratio of total triterpenoid content between cultures for 34 days and 17 days was 2.20 and 1.88, and for 51 days and 34 days was 1.30 and 1.26 in H and L, respectively. The results show that the total triterpenoid content of the two strains was significantly different at different culture times, and the amplitude of increased content of the two strains increased and then decreased over time. The results suggest that the difference of gene expression at different culture times may lead to the difference of synthesis and final accumulation of secondary triterpenoid metabolites.

### 2.2. RNA Sequencing and Assembly Results of Transcriptomes of H and L

The mycelia of H and L were cultured for 17 days, 34 days, and 51 days, repeated three times. An Illumina HiSeq^TM^ 4000 system [23] was used for RNA sequencing. A total of 1,093,590,526 original reads were obtained by sequencing. There were 1,076,215,706 high quality sequences after read adapters, unknown nucleotides, and low-quality reads were filtered (Table 1). Bowtie2 [24] was used to compare high-quality clean reads with reference gene sequences of all samples, and the alignment rate was higher than 92% (Supplementary Table S1). The total high-quality reads was 16,879 unigenes, with an average length of 1602 bp, a maximum length of 16,094 bp, and a minimum length of 201 bp, and N50 is 2673. The quality evaluation of the assembly results can be evaluated from the N50 value. If we sort all unigenes from long to short and add up the length, when the cumulative fragment length reaches 50% of the total fragment length (the length of all unigenes), the corresponding length of that fragment is the length of unigene N50. The longer unigene N50 is, the better the assembly quality. The results of assembly were better than those of Shu et al. (average length 646 bp, N50 = 814) [21] and Zhang et al. (mean length 1114 bp) [18]. The results indicate that the assembly quality of transcriptome sequencing was better in this study. All the sequencing data was submitted to Genbank. The accession number of this project is PRJNA552734.

### 2.3. Principal Component Analysis for Correction of Differentially Expressed Genes (DEGs)

The assembly results were used as the transcription reference gene bank of *W. cocos* and compared with each sample to calculate the gene expression level of reads per kilobase of transcript per million mapped reads (RPKM) [25] in each sample and normalization (Supplementary Table S2). Principal component analysis (PCA) was used to sample the repeatability test according to all gene expressions in each sample (Supplementary Figure S1a). The results of the first principal component indicated that the samples have strong positive correlation in H and L, which were grouped into two categories. Three periods in H were also clustered separately, among which Hd34-2 and the other two repeats were slightly separated in the second principal component. In L, Ld34-2, and Ld51-3 were slightly separated from other two repeats in the second principal component. In order to improve the repeatability between replicates in different periods, PCA was performed again after removing the data of Hd34-2, Ld34-2, and Ld51-3. Subsequent analysis results show improved repeatability between replicates (Supplementary Figure S1b).

To increase the reliability of DEGs between comparison groups, the edgeR function [26] was used to analyze the transcriptome data after removing the data of Hd34-2, Ld34-2, and Ld51-3. The false discovery rate (FDR) [27] and log_2_FC were used as filters, and the filter condition were FDR < 0.05 and |log_2_FC| ≥ 1. According to the statistics of gene expression level (Supplementary Figure S2c), there were 3115 DEGs between H and L at 34 days, among which 2165 were upregulated and 950 were downregulated. There were 2035 DEGs between the Ld17 and Hd17 groups and 2681 DEGs between the Ld51 and Hd51 groups. The number of DEGs increased and then decreased over time, which was consistent with the accumulation of total triterpenoid content in the samples. In the high-yielding strains, there were 264 DEGs between the Hd34 and Hd17 groups, and 568 between the Hd51 and Hd34 groups, showing an upward trend (Supplementary Figure S2b). In the low-yielding strains, there are 1368 DEGs between the Ld34 and Ld17 groups, and 369 between the Ld51 and Ld34 groups, showing a downward trend (Supplementary Figure S2a). In the early stage of triterpenoid content with large differences, the number of DEGs in L was far greater than in H. However, in the later stage when the difference of triterpenoid content decreased, the number of DEGs increased in H and decreased sharply in L, and finally the number of DEGs in H was higher than L. The results show that the differential expression of genes was not completely consistent with the amount of synthesized triterpenes, suggesting that the effect of triterpenes production was not mainly in the transcriptional level of genes, but in the post-transcriptional translation or post-translational modification.

### 2.4. Verification of Transcriptome Sequencing Results by RT-qPCR Experiment

**Figure 2:**
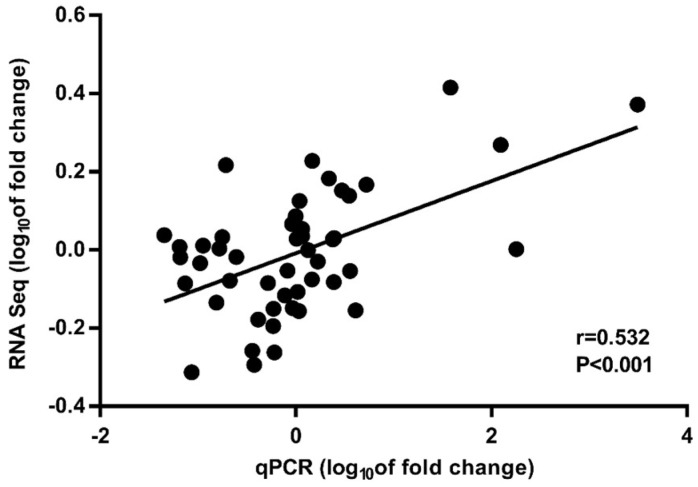
Correlation between RT-qPCR and RNA sequencing of 12 genes. Each point represents a multiple of the expression level at d 34 or d 51, at d 17 or d 34. Change in power is log base 10 transformation.

To verify the reliability of gene expression data obtained by transcriptome sequencing, 12 genes were randomly selected for an RT-qPCR experiment (Supplementary Table S3). The results show that the trend of gene expression was consistent in the transcriptome sequencing and RT-qPCR experiments, and the data presented good correlation (*r* = 0.532, *p* < 0.001; Figure 2). For each gene, the expression results in RT-qPCR showed a similar trend to the data of transcriptome sequencing (Supplementary Figure S3). The results show that transcriptome sequencing was reliable in this study.

### 2.5. Gene Expression Pattern and DEG Function Enrichment Analysis in H and L

**Figure 3:**
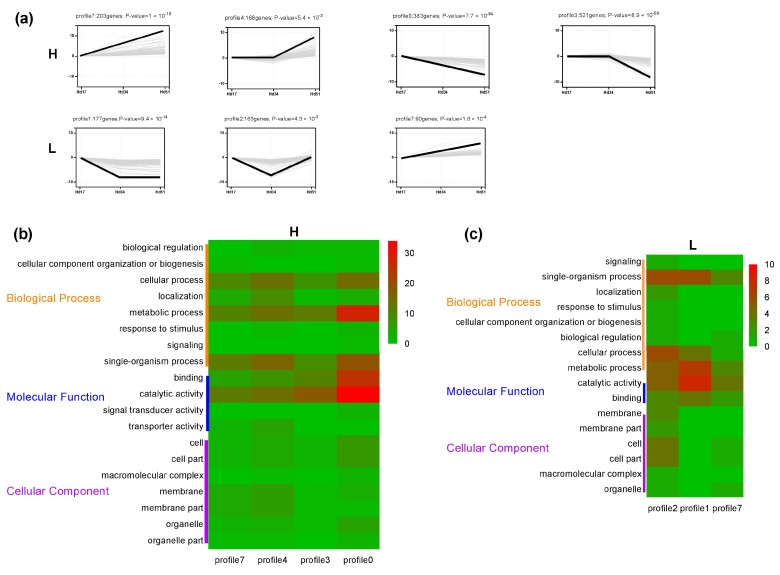
Patterns of gene expressions and analysis of Gene Ontology (GO) enrichment difference of the two strains. (**a**) In each pattern box, the thin gray line represents a gene expressing in this pattern, and the thick black line represents the expression trend of all genes in this pattern. The number of genes for this pattern and its *p*-value are marked on the pattern box. (**b**) Result of GO enrichment of genes of four significantly expressed patterns in high-yielding strains. (**c**) Result of GO enrichment of genes of three significantly expressed patterns in low-yielding strains. The number of genes in each group is represented by color; red means more genes and green means fewer genes.

Short Time-Series Expression Miner (STEM) software [28] was used to analyze gene expression patterns of all DEGs in three culture periods in H and L, and eight gene expression patterns were obtained. There were four significant expression patterns in H (profiles 0, 3, 4, 7; *p* < 0.001), and the opposite expression patterns were present in the same strain at the same time. In the low-yielding group, there were three significant expression patterns (profiles 1, 2, 7; *p* < 0.001), which also reflected the existence of different expression patterns of genes. The significantly opposite expression patterns obtained from the two strains could be divided into two general trends of increase and decrease (Figure 3a).

In order to determine the functional significance of DEGs in the two strains, Gene Ontology (GO) [29,30] and Kyoto Encyclopedia of Genes and Genomes (KEGG) enrichment analysis were performed on the genes with significant expression patterns. It was very interesting that the results of GO enrichment focused on the metabolic process, catalytic activity, cell process, and single tissue process in both strains (Figure 3b,c; Supplementary Tables S4 and S5). These results suggest that the differences in triterpenoid biosynthesis may be directly related to the expression levels and patterns of these genes. The results of KEGG enrichment were different from those of GO enrichment. The genes with significant expression patterns in the two strains all had significantly enriched metabolic pathways for pentose and glucuronic acid conversion in carbohydrate metabolism, and ketone synthesis and degradation in lipid metabolism; but carbohydrate, amino acid, and lipid metabolism pathways of the two strains were different. H had more secondary categories of pathways than L, such as energy metabolism, comprehensive overview, and signal transmission, but less polysaccharide biosynthesis and metabolism and other amino acid metabolism (Supplementary Tables S6 and S7).

### 2.6. Correlation Analysis of Genes Related to Triterpenoid Biosynthesis in W. cocos

Due to the significant differences in triterpenoid metabolites between the two strains, we conducted Venn diagrams for the groups with the opposite expression patterns in H and L (profiles 7 and 4 in H overlap profiles 1 and 2 in L; profiles 0 and 3 in H overlap profile 7 in L) (Supplementary Figure S4a,b). There were 72 genes with opposite expression patterns in both sets. The differential expression of these genes may be responsible for the differences in biosynthesis of triterpenoid between the two strains in *W. cocos* (Supplementary Table S8). GO enrichment analysis was conducted for the 72 overlapping genes (Figure S4c), and it was found that these genes were mainly concentrated in the metabolic, cellular and single-organism processes of biological processes, as well as the catalytic activity and binding of molecular functions. A detailed description of these genes (Supplementary Table S9) shows that biological processes mainly involve single-organism, single-organism metabolic, single-organism cellular, metabolic, cellular, cellular metabolic, and primary metabolic processes. Molecular functions mainly involve catalytic, oxidoreductase, and hydrolase activity; binding, small molecule, ion, organic cyclic compound, and heterocyclic compound binding, and so on.

The 72 genes with significantly opposite expression patterns at three culture times were correlated with the 64 genes annotated to the biosynthesis pathway of terpenoids by transcriptome sequencing in two strains (Supplementary Table S10). Genes with a correlation coefficient r > 0.8 were selected and Cytoscape software was used to make the correlation network diagram (Supplementary Figure S5a). GO enrichment analysis was conducted for the genes in Supplementary Figure S5b, and it was found that these genes were still enriched in the metabolic and single-organism processes in the biological process, as well as the catalytic activity and binding in molecular functions. A detailed description of genes (Supplementary Table S11) shows that they belong to the metabolic, single-organism, single-organism metabolic, lipid metabolism, organic cyclic compound metabolic, organic substance metabolic, primary metabolic, and steroid metabolic processes; catalytic, and oxidoreductase activity; binding, cation, ion, and metal-ion binding; and oxidoreductase activity, acting on CH–OH group of donors; etc.

**Figure 4:**
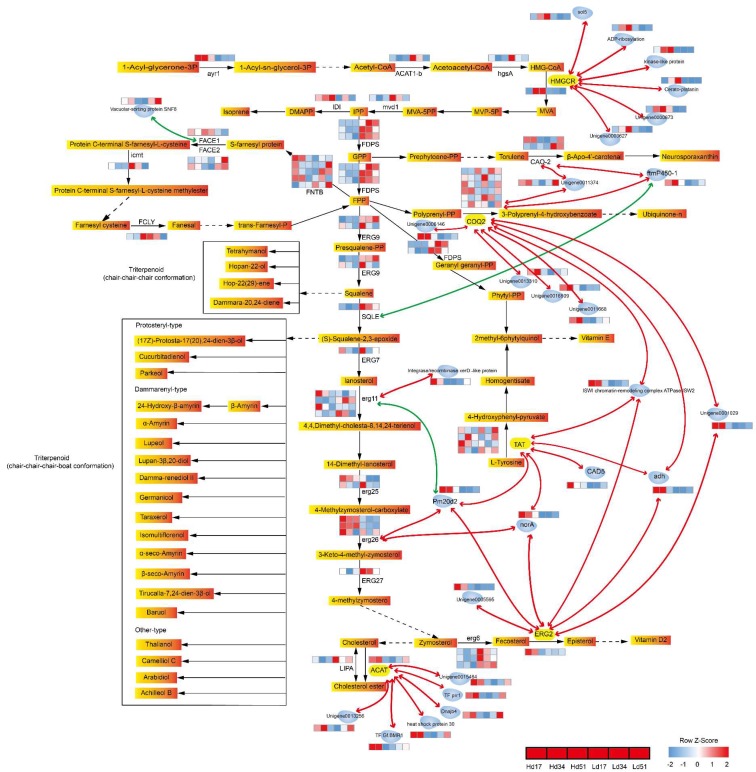
Gene expression differences and relational graph of triterpenoid-related genes. Gradual orange rectangles represent metabolite, enzyme abbreviation, and enzyme expression level of the three time points of the two strains marked on the bottom, left, or right of the arrow connecting two metabolites. Solid black arrows represent direct catalytic reactions, and dotted black arrows represent indirect catalytic reactions. Metabolites in solid black boxes are triterpene products of the same type catalyzed by different enzymes with the same precursor. Blue molecule shapes represent protein genes, marked with gene abbreviation or ID. Red lines represent positive correlation, green lines represent negative correlation, and line thickness represents the correlation coefficient r. The heat map of each gene’s reads per kilobase of transcript per million mapped reads (RPKM) value is z-score standardized.

The genes in Supplementary Figure S5 were mapped to the metabolic pathways associated with triterpenoid (Figure 4, Supplementary Figure S6), where squalene is the precursor of one class of triterpenes and squalene-2,3-epoxy is the precursor of another class. The enzymes catalyzed the synthesis of farnesyl diphosphate (FPP), squalene, and squalene-2,3-epoxy are FDPS, farnesyl-diphosphate farnesyltransferase (ERG9), and squalene monooxygenase (SQLE), respectively. They are highly expressed in the low-yield group, and SQLE is closely negatively correlated with the expression of ftmp450-1. This result is inconsistent with our expectation that high transcription expression of these three genes could improve the production of subsequent secondary metabolites. This suggests that the gene regulation of triterpenoid synthesis may occur at many levels, from transcription to post-translation processing, or the common precursor flows to other branches of non-triterpenes and the upregulation of other branches of genes leads to reduced triterpene production in the low-yield group.

The results of this study show that the genes 4-hydroxybenzoate polyprenyltransferase (COQ2), C-8 sterol isomerase (ERG2), tyrosine aminotransferase (TAT), sterol *O*-acyltransferase (ACAT), and HMGCR are at the core of the triterpenoid biosynthesis pathway. The expression of these core genes is closely related to the expression of multiple protease genes, suggesting that they may be under the regulation of the protease genes. At the same time, several protease genes are closely related to different triterpenoid genes, indicating that they play an important role in the regulation of the core genes of triterpenoid biosynthesis.

HMGCR is an enzyme that catalyzes the synthesis of mevalonate and is upstream of the triterpene biosynthesis precursor. ERG2 and ACAT are enzymes that catalyze the synthesis of episterol and cholesterol ester respectively, and are downstream of the triterpene synthesis precursor. FPP is the precursor of squalene, and can be evolved into ubiquinone and other terpenoid-quinone. COQ2 and TAT are important enzymes for the synthesis of ubiquinone and vitamin E. This indicates not only that the core genes of triterpene synthesis of *W. cocos* include upstream and downstream genes of squalene synthesis but also that branching genes divert the squalene precursor. COQ2, TAT, and ERG2 are connected by multiple protease genes into a complex network and are closely related to erg11 and erg26 in sterol pathways.

## 3. Discussion

In this study, the high-yielding DZAC-Wp-H-29 (H) and low-yielding DZAC-Wp-L-123 (L) strains of *W. cocos* with different total triterpenoid content were screened from the sexual progeny of the same strain. The selection of materials and culture times avoided the background interference caused by different genetic base or developmental stages of materials, making the research results more accurate and reliable. The relationships were emphatically compared among 72 genes with different expression patterns and 64 genes related to triterpenoid biosynthesis in two strains with similar genetic bases. Five core genes related to triterpenoid biosynthesis (HMGCR, COQ2, ERG2, ACAT, and TAT) and seven related to be core proteases gene (Pm20d2, norA, ISWI chromatin-remodeling complex ATPase ISW2, adh, ftmp450-1, and unknown proteins unigene0011374 and unigene0001029) were obtained.

HMGCR catalyzes the conversion of 3-hydroxy-3-methyl-pentanedione-CoA (HMG-CoA) to mevalonate in a four-electron oxidoreduction, which is immobilized in endoplasmic reticulum (ER) with oxidized form of nicotinamide adenine dinucleoside (NAD+) or oxidized form of nicotinamide adenine dinucleotide phosphate (NADP+) as receptor. It is the rate-limiting step in the synthesis of cholesterol and other isoprenoids [31]. HMGCR is a highly conserved membrane-bound enzyme, and its regulation takes place at the levels of transcription, translation, post-translational modification, and degradation [32]. In this study, the HMGCR gene is upregulated in H and is closely related to the expression of flavin-adenine dinucleotide (FAD) binding domain-containing protein, kinase-like protein, cerato-platanin protein, ADP-ribosylation, and other unknown protein genes (Figure 4). Therefore, HMGCR is also an upstream rate-limiting enzyme in the biosynthesis of *W. cocos* triterpenoid and is regulated by various enzyme genes.

ACAT and ERG2 are particularly important among steroid biosynthesis genes closely associated with triterpenoid biosynthesis. ACAT catalyzes esterification of storage of cholesterol and long-chain fatty acids, which are key components of sterol and cell membrane homeostasis [33,34]. ACAT is a membrane protein located in the endoplasmic reticulum [35]. Cholesterol is a regulator of ACAT [34]. Ginsenosides have surface activity, and can combine with sterols in the membrane structure to form precipitation, leading to destruction of the biofilm [36], thus producing certain self-toxicity [37]. In this study, ACAT expression is upregulated in H (Figure 4) and is also closely related to several transcription factors, heat shock protein, and DNA binding-domain-containing protein. The triterpenoid compounds can also be combined with the sterol in the membrane structure to precipitate, leading to destruction of the biofilm, so the stable membrane structure in H needs to synthesize more cholesterol ester. The expression of ERG2 gene is also significantly different between the two strains (Figure 4), and is closely associated with adh, Pm20d2, norA, and ISWI chromatin-remodeling complex ATPase ISW2. These results indicate that ERG2 and ACAT are important rate-limiting enzymes in the triterpenoid synthesis of *W. cocos,* and are regulated by multiple enzyme genes.

TAT and COQ2 are especially important among the ubiquinone and other terpene biosynthesis genes closely related to triterpenoids synthesis. TAT is a key upstream enzyme in the synthesis of vitamin E (Figure 4). In this study, this gene is also closely related to the expression of CAD5, adh, Pm20d2, norA, and ISWI chromatin-remodeling complex ATPase ISW2. COQ2 is a polymeric membrane protein of the UbiA superfamily. The saccharomyces cerevisiae COQ2 gene is required in the biosynthetic pathway of ubiquinone (CoQ). This enzyme catalyzes the prenylation of p-hydroxybenzoate with an all-trans polyprenyl group [38]. The role of terpenoid cyclase as a template for catalysis is paramount to its function. Terpenoid cyclization products of trans-isoprenoid substrates account for the great majority of natural terpenoid products. Linear substrate chain elongation reactions are catalyzed by prenyltransferases [39]. In this study, COQ2 is at the core of the expression network, and is closely related to the expression of nine genes, including adh, ISWI chromatin-remodeling complex ATPase ISW2, ftmP450-1, and unknown genes (Figure 4). These indicate that the regulation mechanism of COQ2 is extremely complex and mysterious. The expression patterns of the six COQ2 genes in two strains are also different in this study, further indicating that the functions and expressions of COQ2 are complex and diverse and need to be studied further.

In the correlation analysis, it was found that seven genes may be important for triterpenoid synthesis, and they are closely related to the expression of genes related to triterpenoid synthesis (Figure 4). Pm20d2 and norA are strongly positively correlated with the expression of TAT, ERG2, and erg26 in terpenoid synthesis, while Pm20d2 is strongly negatively correlated with lanosterol 14-alpha-demethylase (erg11). ISWI chromatin-remodeling complex ATPase ISW2 and adh are also closely positively related to TAT, EGR2, and COQ2 expression. FtmP450-1 is positively correlated with the expression of COQ2 and CAO2, and negatively correlated with a key triterpenoid synthesis enzyme, squalene monooxygenase (SQLE). Thus, the regulation of cytochrome P450 gene plays an important role in triterpenoid synthesis. The unknown protein gene unigene0011374 is closely related to COQ2 and CAO2, and unigene0001029 is closely related to COQ2 and ERG2. The mechanism and function of expression of these genes in the synthesis and accumulation of triterpenoid in *W. cocos* need to be studied further.

FDPS catalyzes isopentenyl diphosphate (IPP) and dimethylallyl diphosphate (DMAPP) to form farnesyl diphosphate, and this reaction is considered to be a rate-limiting step in the biosynthesis of isoprene. *Arabidopsis thaliana* contains at least two genes, FPS1 and FPS2, encoding FDPS, and they have different patterns of expression [40]. In this study, three genes are annotated as FDPS, and their expression patterns are also different, one of which has upregulated expression in L, while the other two genes are not significantly different. It is suggested that the low triterpenoid accumulation in L may not be caused by the low expression of the enzyme genes in the triterpenoid direction, but by the catalytic products of the high expression of the enzyme genes not flowing in the direction of triterpenoid synthesis. The enzymes that catalyze the synthesis of triterpenoids may be regulated in the process of post-transcriptional translation or post-translational modification. The key enzyme gene COQ2, which catalyzes the synthesis of CoQ downstream [41], and the vitamin K cycle of NAD(P)H dehydrogenase gene are highly expressed at the same time in L. These results further indicate that there may be more catalysis product of FDPS than other synthetic approaches, which reduces the synthesis of triterpenoid compound precursor substances, which is the reason for lower triterpenoid compound accumulation in L.

In addition, erg26 is a 3beta-hydroxysterol dehydrogenase that is involved in the removal of C-4 methyl groups in the cholesterol biosynthetic pathway [42]. In this study, two of the three erg26 genes are found to have upregulated expression in H during triterpenoid metabolism, and they are also positively correlated with Pm20d2 and norA expression patterns. It is suggested that these two erg26 genes may also be rate-limiting enzymes of sterol biosynthesis, affecting the accumulation of sterols. Thus, they are also among the key enzymes for the accumulation of triterpenoid.

To sum up, the results of this study suggest that the synthesis and accumulation of triterpenoid in *W. cocos* are influenced by the expression not only of related core pathway genes (HMGCR, FDPS, COQ2, ERG2, ACAT, TAT, CAO2, and erg26), but also of some genes outside the pathway (Pm20d2, norA, ISWI chromatin-remodeling complex ATPase ISW2, adh, ftmp450-1, unknown proteins unigene0011374 and unigene0001029). The difference in triterpenoid synthesis is due not only to the high expression of some genes in the high-yielding strains, but also to the high expression of some genes in the low-yielding strains, which diverts the precursor substances of triterpenoid flow to other channels, resulting in reduced synthesis and accumulation of triterpenoid. To improve the synthesis and accumulation of a certain kind of substance, we need to pay attention to the genes not only with high expression in the high-yielding group, but also with high expression of shunt precursors in the low-yielding group. Controlling their expression may improve the synthesis of required substances. It is also found that a stable membrane structure may be necessary to maintain the high accumulation of triterpenoid in *W. cocos*.

## 4. Materials and Methods

### 4.1. Biomaterials and Culture Methods

Both the high-yielding (DZAC-Wp-H-29) and low-yielding (DZAC-Wp-L-123) triterpenoid strains were derived from the sexually reproduced progeny strains of *W. cocos* 5.78 (purchase from the Institute of Microbiology, Chinese Academy of Sciences, Beijing, China, and stored in a refrigerator at −80 °C at the Institute of Fungal Resources, Guizhou University). For *W. cocos* potato dextrose agar (PDA) medium (no. 17 medium, Institute of Microbiology, Chinese Academy of Sciences), potatoes were washed, peeled, and cut into pieces, and 200 g of potatoes was put into 1000 mL of water, boiled for 30 min, then filtered by gauze. The filtrate was mixed with 1000 mL distilled water with 20 g glucose, 1 g KH_2_PO_4_, 0.5 g MgSO_4_·7H_2_O, 10 mg VB_1_, and 18 g agar at natural pH. Mycelia were cultured for 17 day, 34 day, and 51 day at 25 °C in the dark, quick frozen in liquid nitrogen, then stored in a refrigerator at −80 °C.

### 4.2. Colorimetry Measurement of Total Triterpenoid

Colorimetric determination of total triterpenoid of *W. cocos* was modified by reference to Liu et al. [22]. First, 0.05 g of dry mycelium powder (60 mesh) was placed in a 2 mL centrifuge tube, and 1.5 mL anhydrous ethanol was added. After ultrasonic extraction for 15 min, followed by centrifugation at 10,000 r/min for 5 min, the supernatant was placed in a 5 mL volumetric flask. Then 1.5 mL anhydrous ethanol was again added to the centrifuge tube. After ultrasonic treatment for 15 min and centrifugation at 10,000 r/min for 5 min, the supernatant was taken and merged into the 5 mL volumetric flask, and anhydrous ethanol volume was added to the flask. Then 2 mL of extract was taken in a test tube, volatilized at 50 °C, cooled, and 0.2 mL of 5% vanillin in glacial acetic acid and 1 mL of perchloric acid were added, then they were mixed, bathed in 70 °C water for 20 min, removed and cooled to room temperature, and 5 mL of anhydrous ethanol was added and mixed in. Then 200 μL of mixed liquor was taken for absorbance measurement at 560 nm for 10 to 25 min; the reference substance was oleanolic acid.

### 4.3. RNA Extraction and Quantification Analysis

Because *W. cocos* hyphae are rich in polysaccharides, total RNA was extracted by total RNA extraction auxiliaries and RNAiso Plus (Takara, China Bao Biological Engineering (Dalian) Co., Ltd. Dalian, China), DNA pollution was removed by adding RNase-free DNase I, and three biological repeats were carried out. Total RNA was detected on 1% agarose gel and examined by NanoDrop ND2000 spectrophotometer (NanoDrop Technologies, Wilmington, DE, USA). The RNA integrity number (RIN) values (>8.0) of these samples were evaluated by Agilent 2100 Bioanalyzer (Santa Clara, CA, USA). The purity, concentration, and integrity of total RNA samples were qualified through testing and evaluation, then the samples were prepared for use.

### 4.4. Construction and Sequencing of cDNA Library

First, mRNA was isolated from total RNA with Oligo (dT) beads, then broken into short fragments with fragment buffer. Then short fragments reverse transcripted into the first-strand cDNA with a random primer, and the second-strand cDNA was synthesized with DNA polymerase I, RNase H, dNTP, and buffer solution. The cDNA fragments were purified with 1.8× Agencourt AMPure XP Beads and end repaired, and poly (A) was added and ligated to Illumina sequence adapters. The ligation products were size selected by agarose gel electrophoresis, PCR amplified, and sequenced using Illumina HiSeq^TM^ 4000 by Gene Denovo Biotechnology Co. (Guangzhou, China).

### 4.5. Sequence Assembly and Functional Annotations

Reads obtained from the sequencing machines included dirty reads containing adapters or low-quality bases, which would affect the assembly and analysis. Thus, read adapters, unknown nucleotides, and low-quality reads were removed to get clean, high-quality reads. For filter reads using one’s own scripts, the parameters of data processing steps are as follows: (1) Remove reads containing adapters. (2) Remove reads with N (unknown base) with a ratio greater than 10%. (3) Remove low-quality reads (bases with mass value Q ≤ 20, here accounting for more than 40% of reads). (4) Obtain clean reads.

De novo transcriptome assembly was carried out with the Trinity short reads assembling program [43]. The software parameters were as follows: kmer size = 31, min kmer cov = 12; all other nonimportant parameters were default values. Clean reads were aligned with reference sequences to obtain an alignment rate with Bowtie2 short reads alignment software [24]. The software parameters were the default parameters.

Basic annotation of unigenes includes protein functional, pathway, Cluster of Orthologous Groups of proteins (COG/KOG) functional, and GO annotation. To annotate the unigenes, we used the BLASTx program (http://www.ncbi.nlm.nih.gov/BLAST/) with an E-value threshold of 1 × 10^−5^ priority to the National Center for Biotechnology Information (NCBI) non-redundant protein (Nr) database (http://www.ncbi.nlm.nih.gov), the Swiss-Prot protein database (http://www.expasy.ch/sprot), the KEGG database (http://www.genome.jp/kegg), and the COG/KOG database (http://www.ncbi.nlm.nih.gov/COG). Protein functional annotations could be obtained according to the best alignment results. Finally, ESTScan software [44] was used to predict the coding region of unigenes that could not be compared with the above protein libraries, and the nucleic acid sequence (sequence direction 5′->3′) and amino acid sequence of the coding region were obtained.

GO annotation information of unigenes was analyzed by Blast2GO software according to the Nr annotation information [29], then functional classification of unigenes was performed by WEGO software [30].

### 4.6. Unigene Expression Differential Analysis

Unigene expression was calculated and normalized to RPKM [25]. The formula is

RPKM = (1,000,000 × C)/(N × L/1000)

when RPKM is the expression of unigene A, C is the number of reads that are uniquely mapped to unigene A, N is the total number of reads that are uniquely mapped to all unigenes, and L is the length (base number) of unigene A. Concordant PE read alignments were used to normalize the calculation.

Difference analysis based on edgeR [26] was implemented by the R package. Normalization uses the calcNormFactors function embedded in edgeR. Gene dispersion uses the estimate TagwiseDisp function. Differentially expressed genes were those with FDR < 0.05 and |log_2_FC| ≥ 1. The calculation method of FDR [27] uses Benjamini and Hochberg. The formula is FDR = *p* × (m/k), where *p* is *p*-value, m is the number of inspection, and k is the rank of the inspection *p*-value among all *p*-values (from small to large).

### 4.7. RT-qPCR Validation

RT-qPCR specific primers were designed with Beacon Designer 7.9 (Beijing Biological Technology Co., Ltd. Beijing, China) (Supplementary Table S1). The first strand of cDNA was obtained by reverse transcription with Aidlab’s reverse transcription kit (TUREscript 1st Strand cDNA Synthesis Kit, Aidlab Biotechnologies Co., Ltd. Beijing, China.). RT-qPCR was conducted by using the qTOWER 2.2 PCR System (Jena, Germany) and 2× SYBR^®^ Green PCR Master Mix (DBI). Each reaction was performed in a total reaction mixture volume of 10 μL containing 1 μL of first-strand cDNA as a template. The amplification program was as follows: 3 min at 95 °C and 40 cycles of 10 s at 95 °C and 30 s at 58 °C and 45 s at 72 °C, and finally 10 min at 72 °C. All RT-qPCR was repeated three times, with three technical repeats for each experiment. Expression levels of candidate genes were determined using the 2^−∆∆*C*t^ method. Expression levels were normalized against the reference gene pab1 (unigene0013050).

### 4.8. Expression Trend Analysis

Trend analysis is a method to cluster gene expression patterns (the shape of the expression curve at multiple stages) according to the characteristics of multiple continuous samples (at least three samples that include specific time, space, or processing dose size order). Then the gene sets that match certain biological characteristics, such as continuously increasing of expression, are selected from the clustering results. STEM [28] software was used to input the file of the expression level of all differentially expressed genes (samples were sequenced according to biological logic), and then the parameters –pro 8, ratio 1.0 (the rest are default parameters), were selected for trend analysis. Then the *p*-value of each trend was calculated by the hypothesis test, and the threshold was ≤0.05. The trend block satisfying this condition was defined as the trend of significance.

### 4.9. Statistical Analysis

SPSS Statistics software was used for basic calculation, such as standardization, significance test, and so on. The principal component and correlation analyses were conducted with the R-language package (http://www.r-project.org/). Graph Pad Prism7, Cytoscape3.7.1, and Adobe Illustrator CS6 were used for chart preparation.

## 5. Conclusions

This study explores two new findings: (1) The high accumulation ability of *W. cocos* triterpenoid compound is limited by recognized HMGCR and FDPS gene expression, and is also closely related to COQ2, ERG2, ACAT, TAT, CAO2, and erg26 gene expression in related pathways; at the same time, it is limited by expression of Pm20d2, norA, ISWI chromatin-remodeling complex ATPase ISW2, adh, ftmP450-1, and unknown protein genes unigene0011374 and unigene0001029 outside of pathways. (2) The high accumulation capacity of triterpenoid in *W. cocos* may be related to the synthesis capacity of sterols, and the high expression of key enzymes in the low-yielding strains may increase the synthesis products flowing to other channels, resulting in low triterpenoid accumulation. These new discoveries greatly enrich our basic genetic knowledge of the high-accumulation genotypes of triterpenoid in *W. cocos* and provide important references for molecular-assisted screening and breeding of high-triterpenoid *W. cocos*.

## Figures and Tables

**Table 1:** Throughput and quality of RNA-seq of samples

Sample	No. of Raw Reads	No. of Clean Reads	Q20 (%)	Q30 (%)	GC Content (%)
Hd17-1	49,443,264	48,646,584	99.1	96.98	57.67
Hd17-2	57,069,334	56,143,790	99.09	96.95	57.73
Hd17-3	58,364,474	57,334,282	99.05	96.86	57.42
Hd34-1	47,643,390	46,916,374	99.07	96.9	57.65
Hd34-2	49,995,230	49,215,252	99.08	96.93	57.46
Hd34-3	46,722,588	45,978,738	99.05	96.86	57.46
Hd51-1	52,938,778	52,207,298	99.16	97.16	57.46
Hd51-2	47,556,084	46,827,310	99.08	96.92	57.7
Hd51-3	54,687,348	53,813,252	99.08	96.93	57.48
Ld17-1	49,825,826	48,853,942	99.01	96.75	57.81
Ld17-2	76,163,900	74,830,456	99.06	96.88	57.65
Ld17-3	64,840,360	63,758,552	99.08	96.94	57.72
Ld34-1	69,549,900	68,317,596	99.19	97.38	57.35
Ld34-2	73,145,282	72,046,316	99.22	97.43	57.74
Ld34-3	60,119,712	59,271,498	99.23	97.47	57.65
Ld51-1	74,741,142	73,658,172	99.22	97.44	57.6
Ld51-2	73,090,694	72,050,486	99.23	97.47	57.4
Ld51-3	87,693,220	86,345,808	99.2	97.38	57.79

## Supplementary Materials

Supplementary materials can be found at https://www.mdpi.com/1422-0067/20/15/3703/s1.

## Abbreviations

H	high-yielding strains DZAC-Wp-H-29
L	low-yielding strains DZAC-Wp-L-123
HMGCR	hydroxymethyl glutaryl- coenzyme A reductase
FDPS	farnesyl-diphosphate synthase
COQ2	4-hydroxybenzoate polyprenyltransferase
ERG2	C-8 sterol isomerase
ACAT	sterol *O*-acyltransferase
TAT	tyrosine aminotransferase
CAO2	carotenoid oxygenase
erg26	sterol-4alpha-carboxylate 3-dehydrogenase
Pm20d2	peptidase M20 domain-containing protein 2
norA	aryl-alcohol dehydrogenase
adh	GroES-like protein
ftmP450-1	cytochrome P450
GHs	glycosyl hydrolases
CEs	carbohydrate esterases
AAs	auxiliary activities
CBMs	carbohydrate - binding modules
GTs	glycosyltransferases
PLs	polysaccharide lyases
SSR	simple sequence repetition
MVD	diphosphomevalonate decarboxylase
ERG7	lanosterol synthase
LSD	least-significant difference
DEGs	differentially expressed genes
RPKM	Read Per kb per Million Reads
PCA	principal component analysis
FDR	false discovery rate
qPCR	real-time quantitative polymerase chain reaction
GO	Gene Ontology
KEGG	Kyoto Encyclopedia of Genes and Genomes
HMG-CoA	3-hydroxy-3-methylglutaryl coenzyme A
CAD5	heat shock protein
CoQ	ubiquinone
erg11	lanosterol 14-alpha-demethylase
SQLE	squalene monooxygenase
FPP	farnesyl diphosphate
IPP	isopentenyl diphosphate
DMAPP	dimethylallyl diphosphate
ERG9	farnesyl-diphosphate farnesyltransferase
RIN	RNA integrity number
Nr	non-redundant protein
NAD+	oxidized form of nicotinamide adenine dinucleoside
NADP+	oxidized form of nicotinamide adenine dinucleotide phosphate
FAD	flavin-adenine dinucleotide
PDA	potato dextrose agar
COG/KOG	Cluster of Orthologous Groups of proteins
NCBI	the National Center for Biotechnology Information
