# Supplementary figures and images for: Comparative Analysis of the Characteristics of Triterpenoid Transcriptome from Different Strains of Wolfiporia cocos
Source: Int J Mol Sci. 2019 Jul 29;20(15):3703. doi: 10.3390/ijms20153703 (PMC6696085; doi:10.3390/ijms20153703)

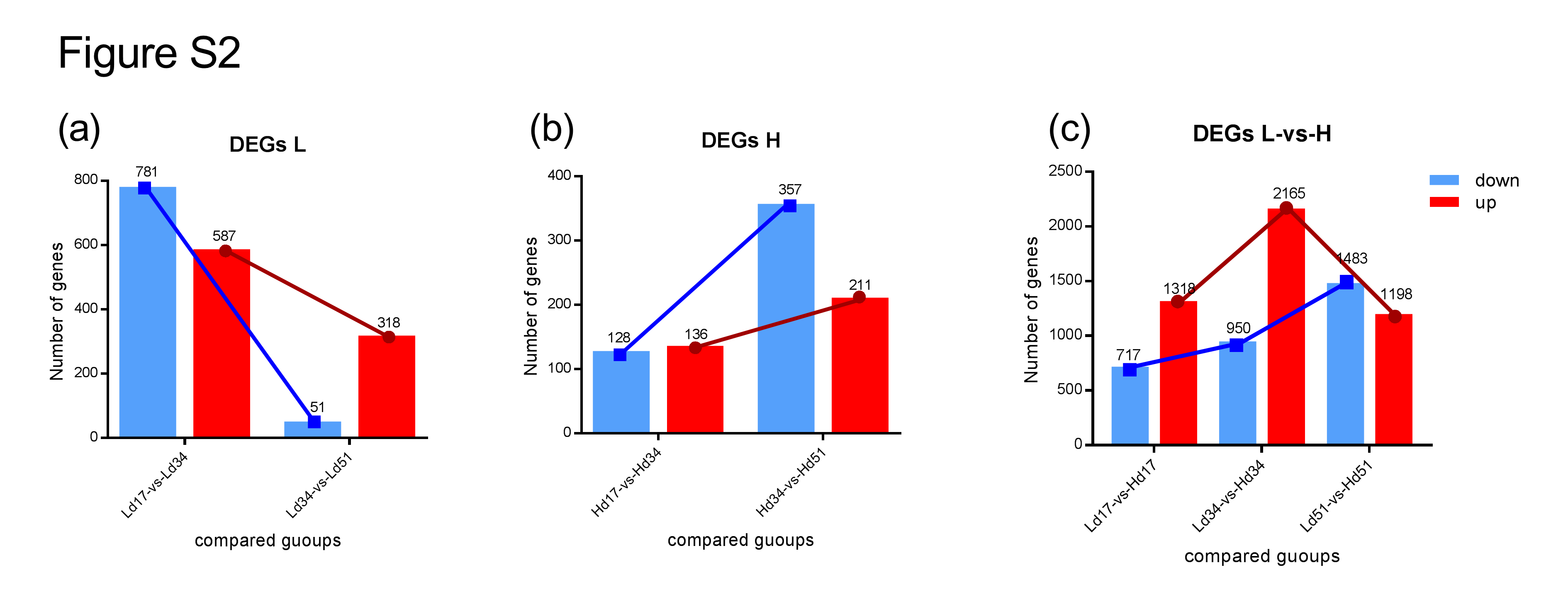

Supplement: Supplementary file 1 [file ijms-20-03703-s001.zip › Figure S2 The number of DEGs..tif]

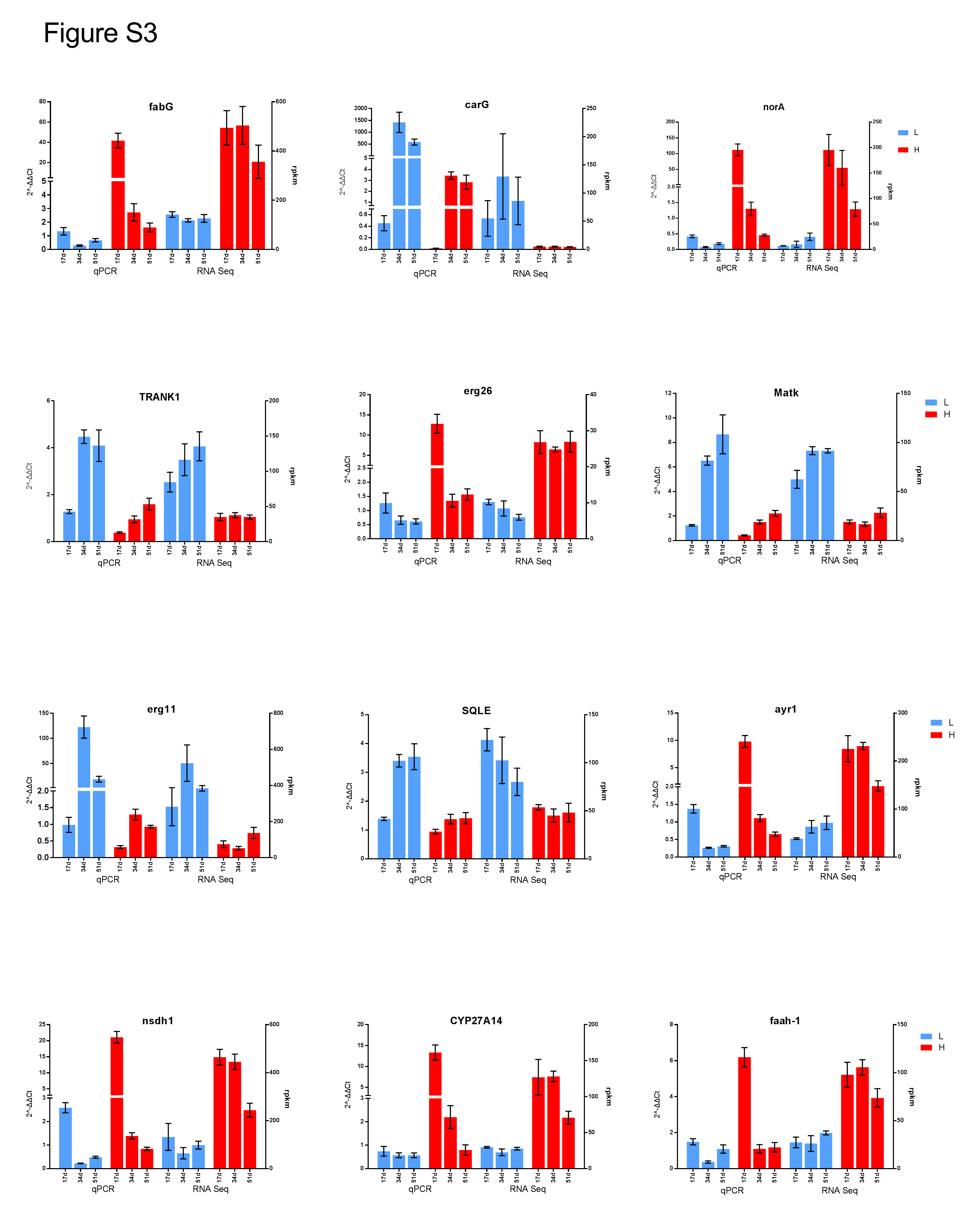

Supplement: Supplementary file 1 [file ijms-20-03703-s001.zip › Figure S3 12 genes expression level of RT-qPCR and RNA sequencing.tif]

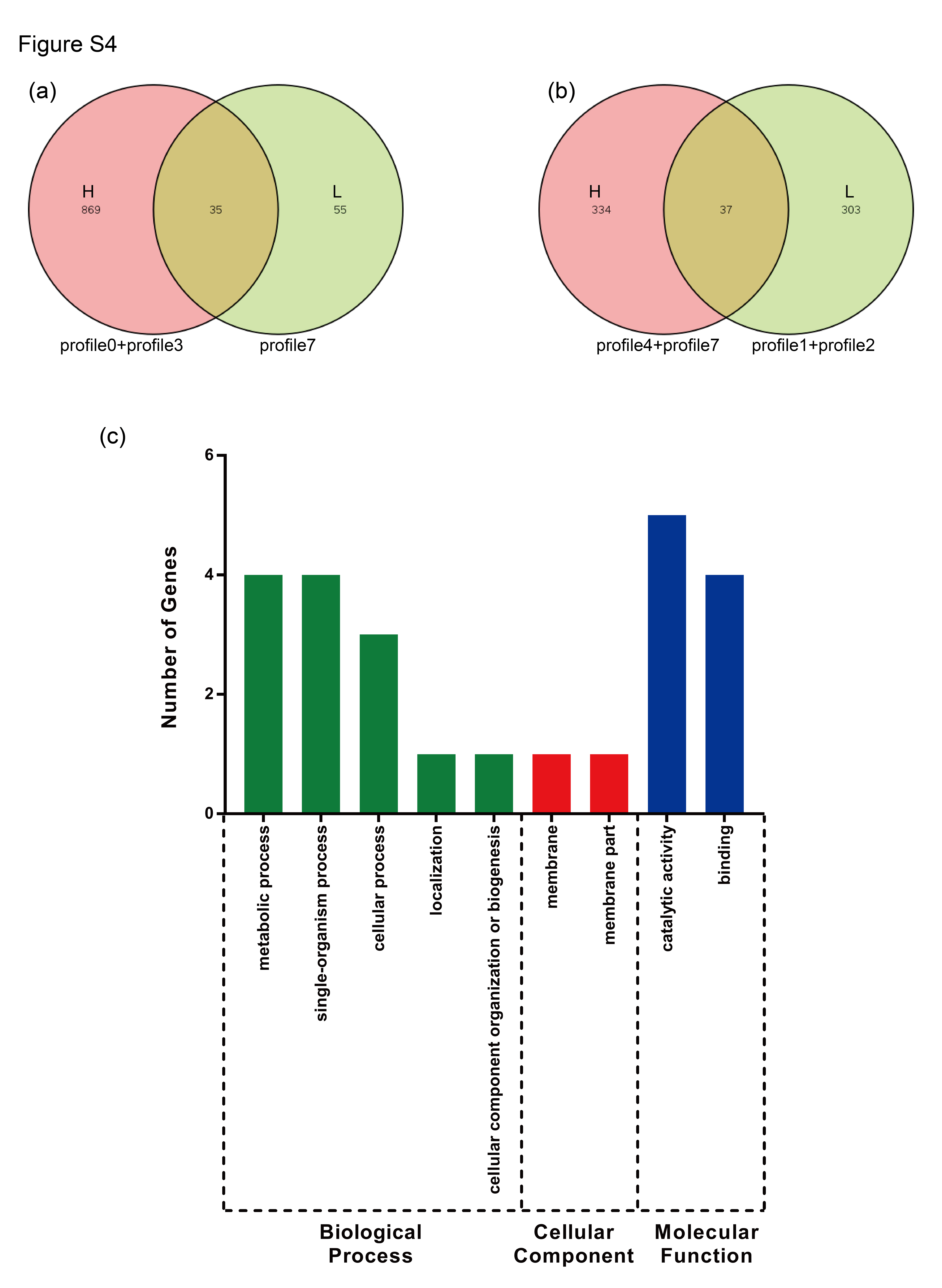

Supplement: Supplementary file 1 [file ijms-20-03703-s001.zip › Figure S4 Venn diagram of genes with opposite expression patterns in two strains..png]

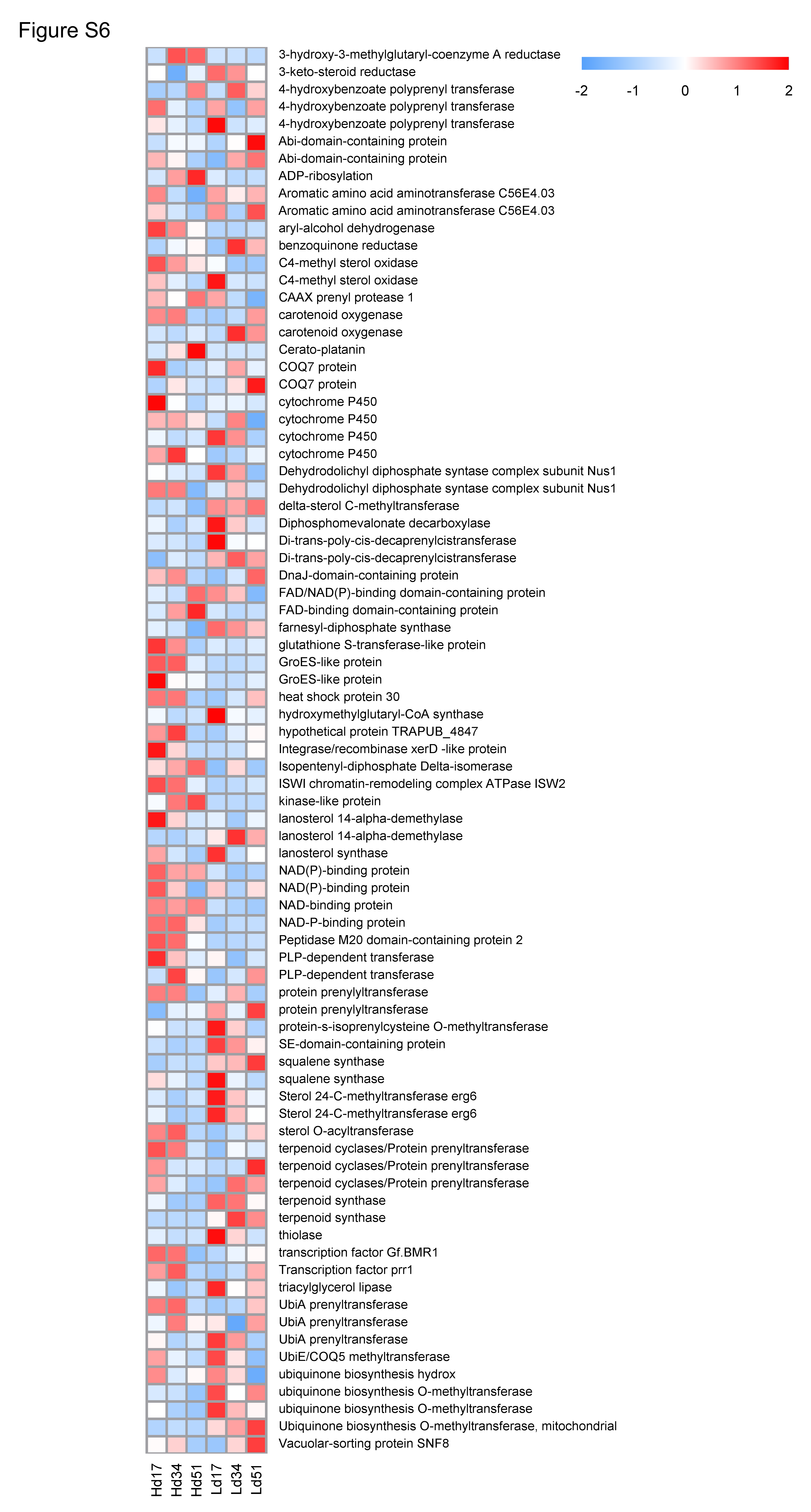

Supplement: Supplementary file 1 [file ijms-20-03703-s001.zip › Figure S6 Standardized heat map of genes in Figure 4.tif]
